# Validity of Danish public criteria for providing flash glucose monitoring to participants with type 1 diabetes—An explorative cohort study

**DOI:** 10.1002/edm2.366

**Published:** 2022-09-15

**Authors:** Ida R. Nielsen, Mie K. Priergaard, Allan Kofoed‐Enevoldsen

**Affiliations:** ^1^ Department of Medicine Nykøbing Falster Hospital and Steno Diabetes Center Nykøbing Falster Denmark

**Keywords:** Danish public regulatory criteria, flash glucose monitor, HbA1c target achievement, predictors, type 1 diabetes

## Abstract

**Introduction:**

Flash glucose monitor (FGM) use is increasing. A set of Danish criteria for regulating the use has been released. We assessed their validity.

**Methods:**

Patients with type 1 diabetes attending our clinic were offered Freestyle Libre Sensor for 12 months and stratified into fulfilling the Danish regional criteria (RC+) or not (RC−). Primary endpoint was achieving individualized target HbA1c. Secondary endpoints were HbA1c reduction ≥5%, time in range (TIR), time below range (TBR), daily scans, change in median HbA1c, and noted experiences.

**Results:**

Two hundred seventy‐eight participants were included. Forty‐four participants met target HbA1c after 1 year. No difference between RC+ and RC− was observed (*p* = .136). Higher age was associated with probability to meet target HbA1c (RR = 3.15, [95% CI: 1.15, 8.62]) as was frequent scans (RR = 1.88, [95% CI: 0.99, 3.57]). One hundred twenty‐three participants met an HbA1c reduction ≥5%, the majority represented in RC+ (*p* = .023). Higher baseline HbA1c was associated with a reduction of HbA1c ≥5% after 1 year (RR = 1.97, [95% CI: 1.40, 2.78]). There was no difference between RC+ and RC− in TIR, TBR, and daily scans. Positive experiences dominated from both participants and healthcare professionals. More positive experiences were noted from healthcare professionals in RC− (*p* = .003) but no difference in reported experiences among participants in RC+ and RC− (*p* = .880).

**Conclusion:**

The Danish Regional Criteria seems not a valid tool for regulation of FGM. Participants of older age and participants with more frequent daily scans might benefit more from FGM.

## Graphical abstract

Use of Flash Glucose Monitoring (FGM) in treatment of diabetes is increasing. We assessed the validity of Danish public institutional criteria for regulatory use of FGM. Our study point out that the seemingly promising current parameters, do not identify the patients with Type 1 diabetes who will benefit from FGM use.

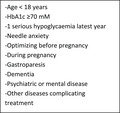

## 1 INTRODUCTION

The demand for sensor‐based blood glucose monitoring is growing globally. Price and limited supply are significant barriers to the spread, and therefore in areas with public or insurance‐based healthcare, institutional regulation of access to sensor‐based measurement may become relevant.

**FIGURE 1:**
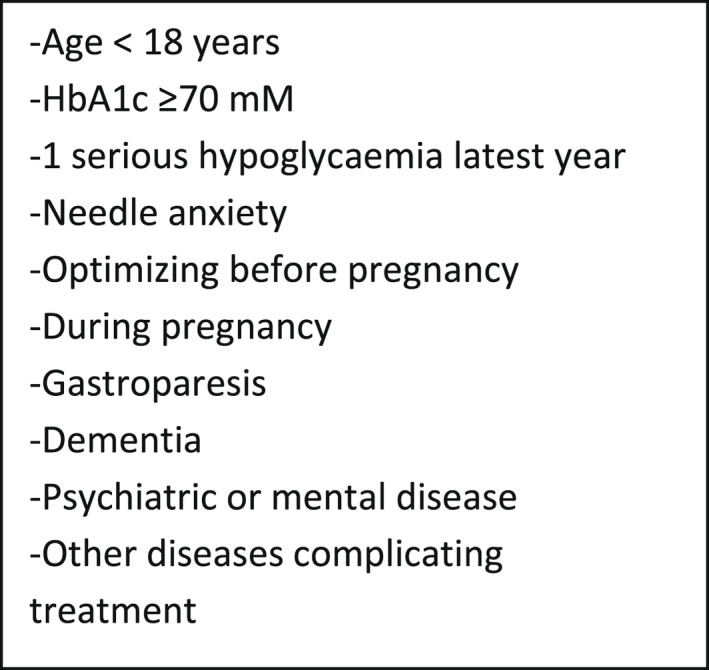
Danish public regional criteria for FGM use in T1DM. HbA1c, Hemoglobin A1c

In Denmark, the public health assurance system covers flash glucose monitors (FGM) to patients with type 1 diabetes (T1DM) as a therapeutic tool. In attempt of regulating the access to FGM, a Danish workgroup approved by the Danish Regional authorities has developed a set of criteria, to be fulfilled in order to receive FGM, in the following referred to as the Regional Criteria (RC; Figure 1).^1^ The validity of these criteria, that is, their ability to identify the patients most suitable for FGM, is unknown. Therefore, we have investigated the validity of these regional criteria through an interventional study in real life settings to see if they are a suitable tool for regulation. Furthermore, we wish to discover any possible aspects, which might predict and find the T1DM patients who will benefit from FGM. To the best of our knowledge, this is the first study to investigate such an attempt of regulation of FGM use.

## 2 METHODS

### 2.1 Study population

Adults with T1DM attending our clinic and considered appropriate for FGM use by any member of the treatment team, were offered Freestyle Libre Sensor (Abbott company) for 12 months and included following consent, not regarding their accommodation to the official Regional Criteria. Participants already using FGM were excluded.

### 2.2 Outcomes

Primary outcome (HbA1c Target Achievement; TA) was improvement of BG regulation, shifting from individual HbA1c target not met at entry to meet after 12 months from inclusion.

The individual HbA1c targets were set as part of standard care by the diabetes team. Individual HbA1c targets were determined and documented by the diabetes team. Most frequently, the targets followed national recommendations: <48 mmol/mol if short diabetes duration and with no complications; <53 mmol/mol if long diabetes duration and with no complications; <58 mmol/mol if significant complications, comorbidity or risk of hypoglycemia; 58–75 mmol/mol if free of symptomatic hyperglycemia was main target. In case of no available registered target HbA1c at entry, a level of at or below 53 mmol/mol was selected for participants with no or only minor late diabetic complications and a level of at or below 58 in case of multiple complications. The outcome was categorized as:

- Grp I: HbA1c target not met at entry was met after 12 months
- Grp II: HbA1c target was met at entry but not met after 12 months
- Grp III: HbA1c remained either within or above HbA1c during the study period

Secondary outcomes were HbA1c reduction ≥5% after 1 year, time in range (TIR), time below range (TBR), number of daily scans, and experience reported by participants and healthcare professionals from patient records.

Sensor based data were extracted from Libreview and Diasend as the mean from 0–1 month, 3–6 months (second quarter), and 9–12 months (fourth quarter) after attachment. Laboratory measured HbA1c was noted from records during study period. The most recently measured HbA1c‐value was noted before FGM attachment and values of HbA1c and dates were noted during the study period until the final HbA1c value within study period. The primary outcome and HbA1c reduction ≥5% were assessed by comparing measured HbA1c to the calculated sensor‐based HbA1c estimate (GMI; Glucose monitoring Indicator) of fourth quarter.

Patient and healthcare professionals overall experience of FGM, as routinely documented in the patient record during the study‐period, were collected and summary classified as either “positive,” “positive and negative,” and “negative.”

### 2.3 Statistics

Statistical analyses were carried out using R vers. 1.1 with *p* < .05 considered significant. To determine if data were normal distributed, visual inspection of histograms and Shapiro–Wilk test were carried out. Continuous variables with normal distribution are presented as mean and standard deviation (SD). Means between two categories were compared by two‐sample *t*‐test and means between three categories were compared by ANOVA. Continuous variables with skewed distribution are expressed as medians and interquartile ranges and were compared by nonparametric tests; Mann–Whitney U test between two groups and the Kruskal–Wallis test between three groups. Counts and percentages were given for categorical variables and compared by the chi‐square test with counts ≥5 and Fisher's exact test with counts <5.

Possible predictors for reaching Target HbA1c and HbA1c reduction were assessed by calculating crude relative risk (RR) with corresponding 95% confidence intervals (95% CI). Cut‐offs for predictors were determined after clinical relevance and homogeneity in groups.

## 3 RESULTS

Five hundred eighteen patients with T1DM had one or more appointments at our clinic during the inclusion period from August 01, 2019 to December 27, 2020. Of these, 278 participants were offered and accepted FGM. The 240 patients not included were either not considered relevant for FGM use, were already using FGM (10%), or declined using FGM. Characteristics of participants are shown in Table 1.

**TABLE 1:** Baseline characteristics of included participants

Characteristics	*N*	Patients
Age	278	49 ± 16
Gender, *n* (%)	278	
Men		159 (57)
Years of T1DM	278	21 [13, 34]
Complications to T1DM, *n* (%)	278	
No complications		110 (40)
A single complication		101 (36)
Multiple complications		67 (24)
HbA1c mmol/mol	275	65 [57, 80]
Education, *n* (%)	229	
Level 1		69 (30)
Level 2		111 (49)
Level 3		49 (21)

*Note*: *Complications to T1DM* were stratified by ICD: International Classification of Diseases and Related Health Problems. No complications: DE109, 109A. A single complication: DE101, DE102, DE103, DE104, DE105, DE105A, DE105B, DE105D, DE106. Multiple complications: DE107*. Educational level* was classified as: (1) No Education, Primary and Lower Secondary Education. (2) General Upper Secondary Education and Vocational Education and Training (3) Bachelor, Professional Bachelor, Academy Profession, Master, Diploma and PhD.

Data are presented as mean ± SD, median [IQR] or count (%).

Abbreviation: T1DM, type 1 diabetes mellitus.

Among the included participants, 157 fulfilled the Regional Criteria for receiving FGM (RC+) whereas 121 did not (RC−). Gender and diabetes duration were equally distributed (Table 2). In RC+, mean age was slightly lower (*p* = .004), multiple complications more frequent (*p* = .023), and educational level lower (*p* = .009). HbA1c was as expected higher in RC+ (*p* < .001).

**TABLE 2:** Baseline characteristics of participants who did or did not fulfil the Danish public regional criteria for receiving FGM

Characteristics	*N*	RC+	*N*	RC−	*p*
Age	157	46 ± 16	121	52 ± 16	.004
Gender, *n* (%)					
Men	157	92 (59)	121	67 (55)	.677
Years of T1DM	157	20 [10, 35]	121	22 [13, 34]	.699
Complications to T1DM, *n* (%)					
No complications	157	54 (34)	121	56 (46)	.023
A single complication		56 (36)		45 (37)	
Multiple complications		47 (30)		20 (17)	
HbA1c mmol/mol	154	76 [64, 89]	121	59 [53, 64]	<.001
Education, *n* (%)					
Level 1	136	49 (36)	93	20 (22)	.009
Level 2		66 (49)		45 (48)	
Level 3		21 (15)		28 (30)	

*Note*: *p*‐values were calculated by two sample *t*‐test, Mann–Whitney U test and chi‐square test.

Data are presented as mean ± SD, median [IQR], or count (%).

Abbreviations: RC+, Participants who did fulfil the Danish public regional criteria. RC−, Participants who did not fulfil the Danish public regional criteria.

### 3.1 Primary outcome

#### 3.1.1 HbA1c target achievement

One hundred eighty‐two participants completed the 12 months follow‐up. Forty‐four improved their BG regulation from individual HbA1c target not met at entry to met after 12 months (TA‐Grp I). No significant difference was observed among the RC+ and RC− groups (*p* = .136), though the share was higher in the RC− group (31% vs. 18%; Table 3). A similar result was found after 6 months (27% vs. 16%, *n* = 216, *p* = .116).

**TABLE 3:** HbA1c Target Achievement of participants who did or did not fulfil the Danish public regional criteria for receiving FGM

	RC+ (*n* = 98)	RC− (*n* = 84)	*p*
TA, *n* (94)			
Grp I	18 (18)	26 (31)	.136
Grp II	5 (5.1)	3 (3.6)	
Grp III	75 (77)	55 (66)	

*Note*: *p*‐value was calculated by Fishers exact test.

Data are presented as count (%).

Abbreviations: Grp I, HbA1c target not met at entry was met after 12 months; Grp II, HbA1c target was met at entry but not met after 12 months; Grp III, HbA1c remained either within or above HbA1c during the study period; RC+, Participants who did fulfil the Danish public regional criteria; RC−, Participants who did not fulfil the Danish public regional criteria; TA, HbA1c Target Achievement.

Characteristics of participants stratified by primary outcome (Table 4) displayed a significantly longer diabetes duration in TA‐Grp I (*p* = .030), as well as a tendency toward lower baseline HbA1c in TA‐Grp II (*p* = .004).

**TABLE 4:** Baseline characteristics of participants associated to HbA1c Target Achievement

Characteristics	Grp I (*n* = 44)	Grp II (*n* = 8)	Grp III (*n* = 130)	*p*
Age	51 ± 16	41 ± 17	47 ± 16	.145
Gender, *n* (%)				
Men	23 (52)	5 (63)	70 (54)	.863
Years of T1DM	28 [16, 42]	16 [7, 29]	19 [13, 29]	.030
Age at T1DM onset	18 [10, 36]	17 [7, 28]	25 [13, 37]	.172
Complications to T1DM, *n* (%)				
No complications	18 (41)	3 (38)	52 (40)	.144
A single complication	14 (32)	1 (13)	55 (42)	
Multiple complications	12 (27)	4 (50)	23 (18)	
Region criteria, *n* (%)				
RC−	26 (59)	3 (38)	55 (42)	.131
HbA1c, mmol/mol	61 [59, 70]	53 [48, 59]	68 [59, 82]	.004

	(*n* = 31)	(*n* = 6)	(*n* = 114)	
Education, *n* (%)				
Level 1	9 (29)	3 (50)	29 (25)	.240
Level 2	18 (58)	1 (17)	57 (50)	
Level 3	4 (13)	2 (33)	28 (25)	

*Note*: *p*‐values were calculated by ANOVA, Kruskal–Wallis test and Fishers exact test.

Data are presented as mean ± SD, median [IQR] or count (%).

Abbreviations: Grp I, HbA1c target not met at entry was met after 12 months; Grp II, HbA1c target was met at entry but not met after 12 months; Grp III, HbA1c remained either within or above HbA1c during the study period; RC+, Participants who did fulfil the Danish public regional criteria; RC−, Participants who did not fulfil the Danish public regional criteria.

### 3.2 Secondary outcomes

#### 3.2.1 HbA1c reduction ≥5%

One hundred twenty‐three participants achieved an HbA1c reduction above 5%, most prevalent in RC+ (76% vs. 58%, *p* = .023). The prevalence of participants increasing their HbA1c above 5% did not differ between RC+ and RC− (12% vs. 14%).

Participants with HbA1c reduction of above 5% had a higher median HbA1c at baseline compared with participants who increased or did not change in HbA1c (69 mmol/mol [IQR: 61, 82] vs. 53 mmol/mol [IQR: 53, 66] and 59 mmol/mol [IQR: 50, 66], *p* < .001).

#### 3.2.2 Time below range, time in range, HbA1c change, and daily scans

**FIGURE 2:**
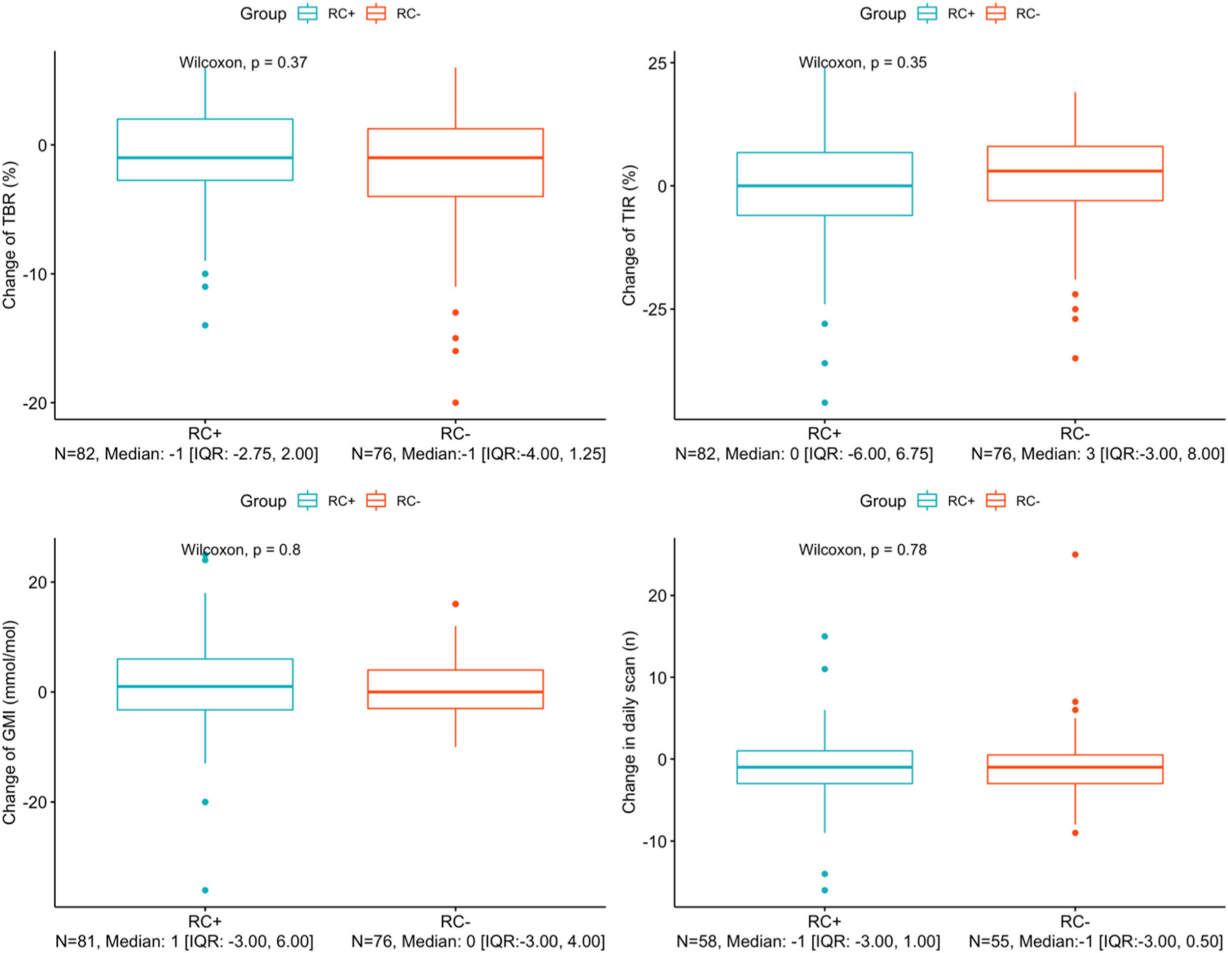
Boxplot of changes of FGM data from the average of 1 month to the average of fourth quarter Each boxplot visualizes the change of Time below Range (TBR%), Time in Range (TIR%), sensor based calculated HbA1c (Glucose monitoring Indicator; GMI mmol/mol), and in number of average daily scan, respectively. The participants were stratified in participants who did (RC+) and did not (RC−) fulfil the Danish Regional Public Criteria for receiving Flash Glucose Monitor. Changes were assessed by comparing the average of 1 month by the average of fourth quarter after attachment. *p*‐values were calculated by Mann–Whitney U test. Data are presented as median [IQR].

Eighty‐two participants in RC+ and 76 in RC− generated sufficient data of TIR and TIL. No significant change was observed during the study period. Also, no change in GMI and daily scan was seen, as well as no difference between the RC+ and RC− groups (Figure 2).

#### 3.2.3 Experience reported by patients

According to the patient record notes, 206 patients expressed their opinion about the FGM experience. The vast majority reported purely positive experiences (Table 5).

**TABLE 5:** Experience by patients and healthcare professionals stratified on participants who did or did not fulfil the Danish public regional criteria for receiving FGM

Experience	RC+	RC−	*p*
	(*n* = 106)	(*n* = 100)	
Patient, *n* (%)			
Positive	100 (94)	96 (96)	.880
Positive & Negative	2 (1.9)	2 (2.0)	
Negative	4 (3.8)	2 (2.0)	

	(*n* = 92)	(*n* = 69)	
Healthcare professional, *n* (%)			
Positive	55 (60)	58 (84)	.003
Positive & Negative	13 (14)	4 (5.8)	
Negative	24 (26)	7 (10)	

*Note*: *p*‐values were calculated by Fishers exact test.

Data are presented as count (%).

Abbreviations: RC+, Participants who did fulfil the Danish public regional criteria. RC−, Participants who did not fulfil the Danish public regional criteria.

#### 3.2.4 Experiences reported by healthcare professionals

Care providers accordingly documented primarily positive experiences, though number of mixed positive/negative or purely negative observations was higher. More positive experiences were reported in RC− (84%) compared with RC+ (60%) and more negative experiences were reported in RC+ (26%, *p* = .003; Table 5). Healthcare professionals reported significantly more positive experiences in elder participants, in participants in RC−, and participants with a lower HbA1c before FGM attachment.

### 3.3 Predictors

Relative risk calculations for possible predictors of TA‐Grp I and HbA1c reduction *≥*5% are displayed in Table 6.

**TABLE 6:** Predictors of reaching HbA1c Target Achievement and HbA1c reduction ≥5%

Predictors	TA‐Grp I		HbA1c reduction ≥5%	
	*N*	RR (95% CI)	*N*	RR (95% CI)
Age				
18–29	4/37	1.00 (ref)	24/37	1.00 (ref)
30–60	24/98	2.65 (0.84, 6.09)	69/98	1.09 (0.83, 1.42)
>60	16/47	3.15(1.15, 8.62)	30/47	0.93 (0.71, 1.36)
Gender				
Women	21/84	1.00 (ref)	56/84	1.00 (ref)
Men	23/98	0.94 (0.56, 1.57)	67/98	1.03 (0.84, 1.26)
Years of T1DM				
≤5	8/27	1.00 (ref)	18/27	1.00 (ref)
6–20	6/63	0.32 (0.12, 0.84)	43/63	1.02 (0.75, 1.40)
>20	30/92	1.10 (0.57, 2.11)	25/92	1.01 (0.75, 1.37)
Complications to T1DM				
No complications	18/73	1.00 (ref)	45/73	1.00 (ref)
A single complication	14/70	0.81 (0.44, 1.50)	54/70	1.25 (1.00, 1.56)
Multiple complications	12/39	1.25 (0.67, 2.32)	24/39	1.00 (0.73, 1.36)
Danish public regional criteria				
RC+	18/98	1.00 (ref)	74/98	1.00 (ref)
RC−	26/84	1.69 (1.00, 2.85)	49/84	0.77 (0.62, 0.96)
Education				
Level 1	9/41	1.00 (ref)	25/41	1.00 (ref)
Level 2	18/76	1.01 (0.53, 2.18)	54/76	1.17 (0.88, 1.55)
Level 3	4/34	0.54 (0.18, 1.59)	21/34	1.01 (0.71, 1.45)
HbA1c mmol/mol				
≤58	11/49	1.00 (ref)	21/49	1.00 (ref)
59–75	27/81	1.48 (0.81, 2.72)	58/81	1.67 (1.18, 2.37)
>75	6/52	0.51 (0.21, 1.28)	44/52	1.97 (1.40, 2.78)
Daily scan				
≤6	12/76	1.00 (ref)	54/76	1.00 (ref)
>6	19/64	1.88 (0.99, 3.57)	41/64	0.90 (0.71, 1.14)
HbA1c change after 1 month				
<5% reduction	2/16	1.00 (ref)	7/16	1.00 (ref)
5%–10% reduction	10/28	2.86 (0.71, 11.45)	19/28	1.55 (0.84, 2.86)
>10% reduction	26/87	2.39 (0.63, 9.09)	76/87	2.00 (1.14, 3.50)
Increase	0/24	0 (0)	3/24	0.29 (0.09, 0.94)

*Note*: *N* = number of participants in TA‐Grp l and who had a HbA1c reduction ≥5% / total number of participants.

*HbA1c change after 1 month* were assessed by comparing HbA1c with the average of GMI of 1 month after attachment.

Age above 60 was associated with a 3 times higher probability of meeting target HbA1c (TA‐Grp I; RR = 3.15, [95% CI: 1.15, 8.62]). None of the 24 participants with increased HbA1c after 1 month met TA‐Grp I after one year (RR = 0, [95% CI: 0]). Belonging to the RC− group or scanning sensor at least 6 times per day displayed a borderline insignificant tendency featuring meeting target HbA1c (TA‐Grp I).

Predictors of HbA1c reduction *≥*5% confirmed the positive effect of baseline HbA1c above 59 mmol/mol. As for the primary endpoint, there were a significantly reduced occurrence of HbA1c reduction ≥5% after 1 year for participants who increased in HbA1c after 1 month (RR = 0.29, [95% CI: 0.09, 0.94]).

## 4 DISCUSSION

To the best of our knowledge, this is the first study to examine the validity of an attempt of public regulation of the access to FGM. This is an explorative cohort study; hence, we did not perform statistical power calculations. We believe, however, that our study is of sufficient strength to validate prospected outcomes by including a relatively large population of 27 participants and long follow‐up time of 12 months.

Caretakers were free to offer FGM to all T1DM patients in care. The intent was to simulate a situation where FGM was not considered a limited resource. We did not intent to enforce the use of FGM in all our T1D patients. Thus, during a consultation, the caretaker needed to decide whether a specific patient could possibly gain from being offered FGM, or not, before offering. This decision was guided purely by the experience and preferences of the caretaker. Thereby, in our opinion, creating the clinically most relevant selection of patients included.

We found no significant association between fulfilling the Regional Criteria and benefitting more from FGM usage. Indeed, HbA1c decrease above 5% did occur more prevalently in the RC+ group, but this group by definition included the highest HbA1c measures and regression toward the mean therefore expected.^2^, ^3^, ^4^, ^5^, ^6^

We are aware of possible bias in our study since our results are partly based on GMI partly HbA1c. We performed a post hoc Bland–Altman plot comparing GMI and HbA1c at time point ±14 days, showing a systematic difference of −6% [95% CI: −8.53, −3.62]. This fixed bias may result in more participants meeting TA‐Grp I but is not likely to obscure any potential benefit of the public Regional Criteria.

We selected individually specified treatment target HbA1c as primary outcome, rather than HbA1c decrease, to avoid the effect of regression toward mean and to acknowledge the value and clinical impact of individual targets. Staff was not aware of the use as primary outcome for our study.

Our study was not designed to demonstrate FGM efficacy of improving blood glucose regulation. We note, however, the lack of change in average TIR, TBR, HbA1c, and number of daily scans, well knowing other studies describes inconsistency and small reduction of HbA1c with FGM.^2^, ^3^, ^4^, ^5^, ^7^, ^8^, ^9^, ^10^

Some patients do improve their glucose levels on FGM. Identifying patients who reached target HbA1c and patients with significant HbA1c decline enabled us to search for predictors of benefit. We saw evidence of effect from high number of daily scans, old age, and long diabetes duration. These findings are in agreement with Tyndall et al. reporting a larger reduction of HbA1c among FGM users diagnosed at a younger age and Dunn et al.^11^ reporting a larger reduction of HbA1c with more frequent scans. Interestingly, we found that increased GMI after 1 month was a strong predictor of not meeting target HbA1c after 1 year. Hence, patients who increase in GMI after first month of FGM usage might represent attention for care improvement, or very unlike to meet HbA1c target within the forthcoming year.

The majority of patients and healthcare professionals expressed positive experience in FGM use. Independently, as it seems, of whether targets are reached or not or significant changes in HbA1c are seen. This observation is probably what ensures a steadily growing demand.

Our study leaves only little positive evidence for specific parameters to be chosen in future public regulatory initiatives. But it does point out that the seemingly promising current parameters, do not identify the patients with type 1 diabetes who will benefit from FGM use.

## AUTHOR CONTRIBUTIONS

Allan Kofoed‐Enevoldsen contributed to the experimental design, discussion and review of the manuscript and researched data. Mie K. Priergaard contributed to the experimental design, discussion and review of the manuscript and statistical analysis. Ida R. Nielsen contributed to the experimental design, discussion and wrote the manuscript, researched data. Ida R. Nielsen is the guarantor of this work and, as such, had full access to all the data in the study and takes responsibility for the integrity of the data and the accuracy of the data analysis.

## FUNDING INFORMATION

This study received grants from the Department of Medicine, Nykøbing Falster Hospital and Steno Diabetes Center, Zealand.

## CONFLICT OF INTEREST

The authors declare no conflicts of interest.

## Data Availability

The data that support the findings of this study are available from the corresponding author upon reasonable request.
